# Authorship network bias in meta-analysis

**DOI:** 10.1017/rsm.2025.10063

**Published:** 2026-02-18

**Authors:** Marvin Rieck, Anne-Christine Mupepele, Carsten F. Dormann

**Affiliations:** 1 Department of Biometry and Environmental System Analysis, University of Freiburg, Germany; 2 Animal Ecology, Department of Biology, University of Marburg, Germany

**Keywords:** author dependence, authorship influence, authorship network bias, collaboration network, meta-analysis, non-independence

## Abstract

1. Meta-analyses are a reliable method for a quantitative research synthesis. They are, however, prone to specific biases that can be introduced in the process. Such a bias could exist if primary literature produces similar results if coming from the same authors. Authorship network bias is the non-independence of effect sizes introduced by the overlap of authors of primary studies. If not accounted for, it can severely impact the quality of meta-analysis and the conclusions drawn from it.

2. To account for such non-independence, multilevel models with author clusters as an additional hierarchy level were recently suggested. We propose a new method for the detection of non-independent effect sizes based on authorship networks and for their correction.

3. An analysis of simulated data demonstrates the effectiveness of the here-suggested new method. We further applied our new method to nine exemplary meta-analyses.

4. Our new method for detection and effective correction can be easily integrated in existing meta-analysis workflows, using the functionality already offered by R’s metafor package.

5. Our goal is to enhance the reliability of meta-analyses by highlighting potential authorship network bias and offering a method to address this often-overlooked bias.

## Highlights

### What is already known?

Authorship network bias is one of many specific biases that can be introduced when conducting a meta-analysis. Multilevel modelling has been proposed as a method for dealing with authorship network bias.

### What is new?

We propose a method for the correction of authorship network bias that circumvents the main problems that the multilevel approach faces. Instead of adding an additional hierarchy level in the model, we use pairwise distances of papers from an authorship overlap network to account for the similarity of effect sizes.

### Potential impact for RSM readers

The presented method is suitable for any meta-analysis dealing with quantitative outcome measures. It can be readily used by researchers within the framework of R’s metafor package.

## 1 Introduction

Research syntheses play an important role in scientific knowledge gain. They synthesise primary literature and provide the best evidence, according to evidence-based practice,^1^ and allow for a generalisation of findings in comparison to primary studies.^2^ ^,^ ^3^ Research syntheses need to be based on systematic review standards and are at best based on a quantitative synthesis and include a formal meta-analysis. To achieve a high standard, the primary studies contributing to a meta-analysis must be based on reasonable and objective inclusion criteria and not rely on the meta-analyst’s preferences, ideas, or opinions. Inadequate selection of inclusion criteria and researcher-dependent choices can lead to biased results.^4^

Even with carefully chosen and unproblematic selection criteria, there are other potentially influential biases specific to meta-analysis, such as indexing bias, search bias, reference bias, and publication bias.^5^ To prevent bias from distorting results, methodological approaches have been developed to support the detection of biases, e.g., funnel plots to detect a publication bias.^2^

Bias can also be introduced by prior beliefs of researchers that shape every step in research from finding a topic of interest to formulating hypotheses, collecting data, and conducting experiments, the analysis or how results are finally interpreted and communicated. In a crowd-sourced analysis, differences were found between researchers due to quantitatively correct yet subjective methodological choices.^6^ Similarly, Singh et al. (2013)^7^ and Munder et al. (2012)^8^ found significant authorship effects on the outcome of therapy assessments. While other factors, such as conflicts of interest, may play a role, some of this authorship effect can likely be attributed to what the analysts *believe to know* about the topic beforehand. If such knowledge is correct, it would be labelled as ‘expertise’, otherwise as ‘prejudice’. Detaching oneself from all prior knowledge or belief is undesirable as it would ignore our state of knowledge, but if prior belief leads to a loss of variation within researcher groups, relative to the variation between research groups, it should be accounted for when synthesising data.

In meta-analysis, data (i.e., effect sizes estimated from primary studies) are commonly analysed based on the assumption that data points are independent.^2^ ^,^ ^9^ ^–^ ^11^ There are however dependencies based on, e.g., geographic or phylogenetic proximity. If these dependencies are ignored, they can strongly influence the conclusion of meta-analyses.^12^ It is, by now, common practice to account for non-independence, e.g., by taking into account phylogenetic proximity when analysing effects across multiple species.^13^ ^–^ ^15^

Non-independence of data can also occur due to methodological differences, different research traditions and schools of thought, influence of heads of labs, supervisors, or eminent scientists and they *could* all lead to biased results in meta-analyses.^10^ ^,^ ^16^ ^–^ ^19^ There is an increasing awareness of this potential source of bias. Proposed remedies include unspecific suggestions, such as ‘pay[ing] more attention to the issue’,^20^ assessing the influence of potentially biased single authors on a study outcome,^7^ or estimating author(team)-specific outcome measures and comparing them to the overall meta-analysis result.^21^ These approaches do not take into account a potential bias spillover between papers and authorship network structures. If two papers share one or more authors, they have an authorship overlap and can be considered connected. Integrating an authorship overlap in meta-analysis goes further than previous approaches in which data dependencies have been considered only if they were strictly the same, e.g., the same author, same lab, without considering partial overlap.^16^ Hierarchical dependence models have been used to account for data dependencies in general,^9^ ^,^ ^10^ ^,^ ^22^ and such models also have the potential to be used for authorship network bias.^17^ This approach is easily incorporated into the existing framework of multilevel models and the interpretation of results is relatively straightforward. It does, however, have some limitations in regard to sample sizes in the higher hierarchy levels and authorship network structure. Here, we present an approach that uses hierarchical models to consider non-independence in authorship as gradual rather than clustered, providing a more versatile and realistic representation of authorship networks.

The goal of this research is to establish a new method to detect and account for an authorship network bias. We first introduce the general concepts behind an authorship overlap and explain the new method involving three steps: 1. detecting potential authorship bias; 2. correcting for the authorship bias; and 3. verifying whether the bias correction was successful. Next, we use simulations of authorship networks to demonstrate that the proposed approach works. At last, we apply the approach to previously published meta-analyses. We also consider potential further developments of the method, discuss its limitations, and compare it with other existing methods as well as providing advice on when to use our suggested approach. Appendix C of the Supplementary Material provides a code-based step-by-step tutorial of how to reanalyse one of the previously published meta-analyses: Gibson et al. (2011).^23^

## 2 Methods

### 2.1 Authorship networks

Authorship overlap can be represented by networks as well as by a similarity matrix, where 0 indicates no overlap (= no link). Authorship bias correction is useful only under two conditions: first, that there is an authorship network with at least some links and second, that there is variance in effect sizes. The potential for authorship network bias gains relevance with increasing controversy (i.e., variance in effect sizes) about the topic to be analysed, because it is this variance of effect sizes that this method aims to help explain. If experimental or observational consensus is high and results are largely similar, then there is little variance to explain by authorship overlap of studies in the first place, and hence, the idea of more closely related papers leading to more similar results is somewhat obsolete. A more detailed look at which parameters are especially influential is given in Section 3.1.

### 2.2 Detection of authorship network bias

**Figure 1:**
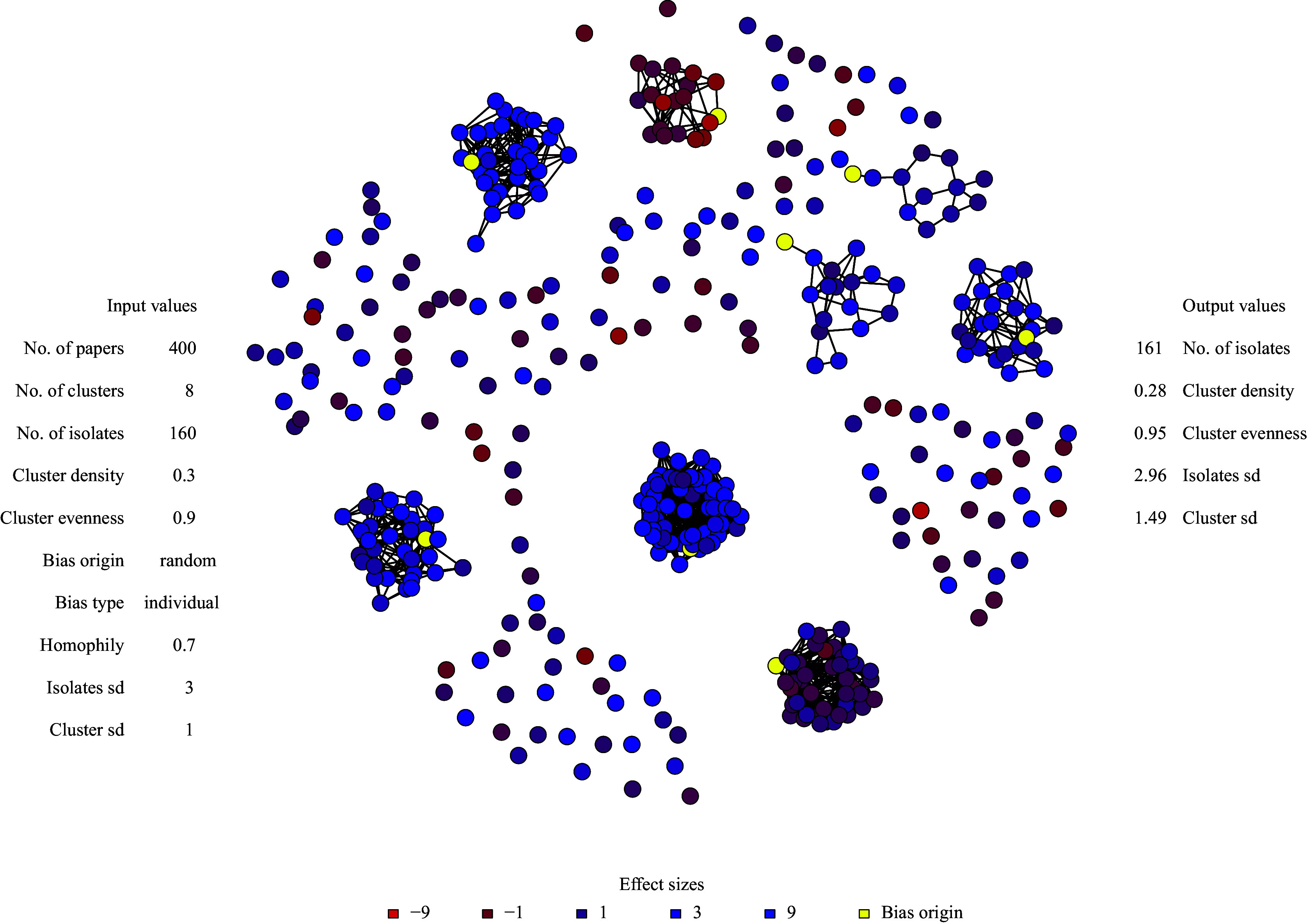
Example of simulated authorship network as analysed in Section 3.1. See Table 1 for input and output parameters and their explanation. Figure 1 Long description.

**Figure 2:**
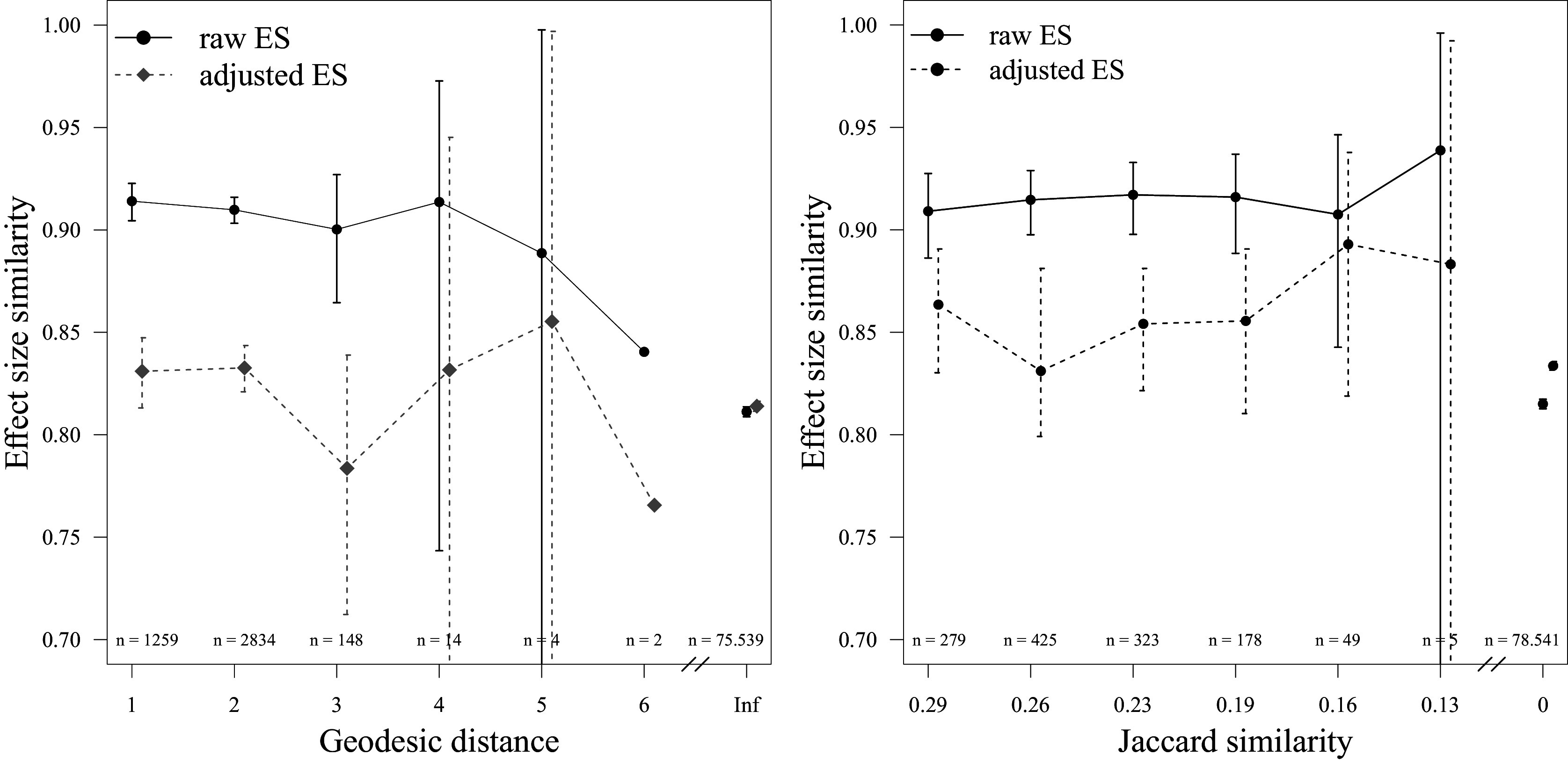
Correlograms of simulated network with authorship network bias. The left panel shows raw and adjusted effect sizes (ESs) for the geodesic distance model. Because of too few data, no confidence interval could be calculated at geodesic distance =6 . The right panel shows raw and adjusted ESs for the Jaccard model. Note that Jaccard distance ≠1 can only occur for paper pairs with geodesic distance 1. Jaccard distance was calculated by subtracting Jaccard similarity from one. Note that the CI width is proportional to the number of pairwise distances in the respective distance bin. Wide CIs are less informative of the correction outcome but are also less important for assessment because of their low number. Figure 2 Long description.

For the detection of potential authorship network bias, we calculate the distance between primary studies based on authorship overlap. In principle, any kind of distance measure could be used. Here, we use two conceptually complementary measures of paper authorship separation, i.e., *geodesic distance* (the length of the shortest path between two studies in the authorship network; Figure 1) and Jaccard distance (calculated by subtracting Jaccard similarity from one). Conceptually, geodesic distance reflects the structure of the authorship network well, but does not differentiate between small and large proportions of shared authors. To reflect the proportion of shared authors, Jaccard distance can be used additionally. Because the Jaccard distance alone cannot cover network structures, it complements rather than replaces geodesic distance. A drop of effect size similarity with increasing distance of papers indicates authorship network bias (Figure 2).

Effect size similarity is calculated by computing all pairwise differences between two effect sizes *y* in the meta-analysis. This results in an n×n matrix of effect size differences, $\Delta_{\text{ES}}$ , which we then range between 0 and 1. For a matrix of effect similarities, $S_{\text{ES}}$ , rather than differences, this value is subtracted from 1:

(1) $$\begin{array}{rl} \Delta_{\text{ES}}(i,j) & =|y_{i}-y_{j}|\\ S_{\text{ES}}(i,j) & =1-\frac{\Delta_{ij}}{\max(\Delta )}. \end{array}$$

Plotting effect size similarity against Jaccard or geodesic distance, which is reflecting the authorship overlap, results in a correlogram (Figure 2). A horizontal line from geodesic distance one to infinity would mean that authorship network distances do not influence similarities between effect sizes. Decreasing effect size similarity with higher geodesic distance values, and/or a high difference of effect sizes between finite and infinite geodesic distances, indicate that effect sizes are influenced by authorship overlap.

### 2.3 Correcting for authorship network bias

If authorship network bias was detected, the ensuing analysis is equivalent to a phylogenetic meta-analysis.^12^ Instead of a phylogeny, we construct a similarity matrix $\Sigma_{\text{AN}}$ , based on (normalised) pairwise geodesic distances $\Delta_{ij}$ between two studies *i* and *j*. Next, we compute the effect-size similarity among unconnected studies in the authorship network, i.e., the similarity at distance infinity or authorship network weighting factor $S_{\infty }\;=\;\;1-\;{\hat{\Delta }}_{\text{between cluster}}$ . This is then used to construct a weighted authorship network similarity matrix:

(2) $$\begin{array}{r} W_{\text{AN}}(i,j)=1-S_{\infty }\cdot \frac{\Delta_{ij}}{\max(\Delta )}. \end{array}$$

The value of $S_{\infty }$ determines the influence of the geodesic distance matrix: if even independent studies differ little in their effect sizes, then $S_{\infty }$ is close to one and authorship overlap bias ( $\frac{\Delta_{ij}}{\max(\Delta )}$ ) cannot have a strong influence on the results. If, however, differences are substantial, then $S_{\infty }$ is low, and the potential effect of authorship overlap is high, resulting in more similar results among studies of an authorship cluster and an overall wider range of possible values in the similarity matrix $W_{\text{AN}}$ .

The model used to estimate adjusted effect sizes while accounting for an authorship network bias is based on a Generalised Least Squares approach, where the similarity matrix $\Sigma_{\text{AN}}$ is provided as variance–covariance matrix.^14^ In the context of meta-analysis, it is internally multiplied by the study-specific variances, a procedure implemented in the metafor package for R.^24^ The constructed similarity matrix can be used alongside other commonly used specifications of meta-analytical models, such as nesting multiple effect sizes within studies or multiple studies within regions. Even multiple similarity matrices may be used together to account for authorship bias and the phylogenetic relationships between different species.^25^ To reduce type I error rates while keeping constant the chance of committing a type II error, the similarity matrix $\Sigma_{\text{AN}}$ should be included in the model even if its contribution to explaining the variance of the fitted model is small. Cinar et al. (2021)^26^ recommend a similar approach to account for dependent effect sizes of multiple species in phylogenetic meta-analyses. If similarity matrices were included in models in which they did not explain any variance at all, there was no effect on either the reported estimates or their confidence intervals (see ?? for the corresponding code). Thus, in case of doubt, the similarity matrix can simply be included in the analysis, as it presents no disadvantage.

To assess the degree to which the similarity matrix $\Sigma_{\text{AN}}$ mitigates authorship network bias, the adjusted effect sizes can be represented by a post-correction correlogram. To do so, the best linear unbiased predictor (BLUP) of the similarity matrix is subtracted from the raw effect sizes. Now, a correlogram can be created to display the adjusted effect sizes in relation to the geodesic distance and to compare it with the raw effect sizes. Ideally, the resulting line should now be horizontal and the effect sizes similar at finite and infinite geodesic distances. An example of an adjusted effect sizes correlogram is shown in Figure 2. See Section 3.1 for detailed analysis.

The geodesic and Jaccard similarity matrices (equation (1)) can now be included in the meta-analytical model. To take into account both the network structure and the number of shared authors as edge weights, the two matrices can be summed and their values normalised. A unique study identifier column is included as a random effect to which the similarity matrix is assigned. The model summary now gives an estimate corrected for similarity of authorship. Comparing this estimate to the estimate from the model without bias correction gives an idea of how influential the matrix is. More important, however, is that we check a new correlogram of adjusted effect sizes for the pattern observed before.

## 3 Application of the method

### 3.1 Simulation studies

#### 3.1.1 Simulation of authorship networks

Before applying the method outlined above to real-world meta-analyses, we used simulations to explore the potential bias of an authorship network.

We simulated networks with varying effect-size differences (which determine how much bias there could be) and network connectedness (which sets the degree of non-independence). Additionally, other variables, e.g., number of papers and bias size, were included in the simulation of networks as they potentially influence authorship network bias (Table 1). Simulation aimed at identifying single as well as combinations of variables that may have a particular influence on the authorship bias correction approach (see Section 2 and ?? for the complete code for network simulation; see also Appendix A of the Supplementary Material for how data were simulated conceptually).

**Table 1:** Parameters used for network simulation, as well as the respective value for the analysed exemplary network Table 1 Long description.

		Example	Simulation	Observation
Parameter	Explanation	value	range	range
No. of papers	For the simulation, this is the same as the number of effect sizes	400	20–1,000	10–457
No. of clusters	Number of disjunct authorship clusters	8	1–20	2–52
No. of isolates	Number of non-connected papers	160	20%–80%	40%–60%
Cluster density	Proportion of all possible connections in a cluster/likelihood of any two cluster members to be directly connected	0.3	0.2–1	0.79–0.95
Cluster size evenness	Shannon Evenness of clusters (instead of species)	0.9	0.5–1	0.54–1
Bias size	Standard deviation of normal distribution from which bias origin values are drawn	5	-	-
$\sigma_{\text{cluster}}$	Standard deviation of normal distribution around bias origin from which rest of cluster values are drawn	1	-	-
Bias origin	One of: most *central*, *marginal,* or *random* cluster member	*random*	-	-
Bias type	Either whole cluster has same bias (*cluster*) or decreasing influence of bias origin with increasing geodesic distance (*individual*)	*individual*	-	-
Homophily	Sets influence of bias origin on rest of cluster (0–1)	0.7	-	-
$\sigma_{\text{isolates}}$	SD of normal distribution from which values of non-connected papers are drawn	3	-	-

*Note*: Simulation range gives the values that were used in exploring the parameter’s influence on authorship network bias. Observation ranges are the values found in the published meta-analyses that were re-analysed. For cluster density, the mean cluster densities of the respective networks are given. Note that some parameters were only used to construct networks and not extracted from existing ones.

Authorship networks with the potential to bias the summary effect size are characterised by three main properties: 1. large effect size differences between studies or clusters of studies (potential for large bias); 2. high connectedness: a large proportion of studies have at least some overlap with other studies, enabling potential bias spillover; and 3. a pronounced clustering in the network containing bias spillover in separate clusters, making cluster differences more pronounced than they would be in an evenly connected network.

For our simulation, we tested low to high bias scenarios as well as low to high inter- as well as intra-cluster similarity of effect sizes. Intra-cluster effect size similarity was set by $\sigma_{\text{cluster}}$ , homophily, and cluster density, while inter-cluster effect size similarity was mainly controlled by bias size. After an initial exploration phase, bias origin and bias type were kept at the example values for all further analyses because they best reflect real authorship networks.

Network connectedness is mainly driven by how many papers show no overlap (no. of isolates), the number of clusters, and the connectedness within clusters (cluster density). Cluster size evenness was set to 0.9 for most simulations. This is, with some exceptions, reflective of the meta-analyses we reanalysed.

The degree of clustering depends to some extent on the above parameters but was mainly controlled by the number of clusters, their size, and respective density. Cluster density in real meta-analyses was mostly substantially higher than our simulation values, potentially exacerbating bias risk.

$\sigma_{\text{isolates}}$ was mostly set to lower values than $\sigma_{\text{cluster}}$ to imitate “natural” variability of outcomes from which $\sigma_{\text{cluster}}$ deviates. Network size (number of papers) was set to 400 for sensitivity analysis (Figure 4) which is above network sizes in most real meta-analyses. Prior tests showed a non-significant effect of network size on the methods efficacy.

To assess the likelihood of encountering bias in real authorship networks, three metrics should be assessed: effect size differences $\Delta_{\text{ES}}$ (see the equations in Section 2.2). In the following, we will also use connectedness, calculated as in Krackhardt (1994)^27^ and, as a measure of how clustered a network is, the *global clustering coefficient* (GCC) of Wasserman and Faust (1994).^28^ A high GCC means that cluster members are well-connected, facilitating bias spread within clusters. Conversely, in sparsely connected clusters, bias transfer is less likely. However, interpreting GCC requires context: it excludes isolates and two-paper clusters, so a meaningful interpretation can only be achieved if also considering information on the general network structure such as connectedness.

#### 3.1.2 Detection and correction of authorship network bias in simulated networks.

Central to detection and accounting for an authorship network bias is the calculation of the authorship similarity matrix. To do that, we first create a correlogram and extract the mean effect size similarity for the most dissimilar node pairs. In the case of geodesics, this would be at distance infinity; for Jaccard distance, it would be at one. Figure 2 shows the correlograms for geodesic and for Jaccard distances, depicting some within-cluster correlation between geodesic distance and effect size similarity for the raw effect sizes. Importantly, there is a substantial difference between the similarity values of finite and infinite geodesic distances. The latter is also true for the Jaccard correlogram, although no pattern is visible for the within-cluster correlation. Note that the within-cluster correlation for Jaccard applies only to direct neighbours in the network, so patterns in the Jaccard correlogram describe the relationship between the proportion of authorship overlap and effect size similarity, not taking into account indirect neighbours and the general network structure.

A less clear pattern is observable in the geodesic distance plot for adjusted effect sizes and not much change for Jaccard. For both correlograms, however, there is much less difference between unconnected than connected paper pairs. That is, the line from finite to infinite geodesic distance is rather horizontal after authorship similarity correction, meaning the effect size difference is no longer a function of paper similarity.

**Figure 3:**
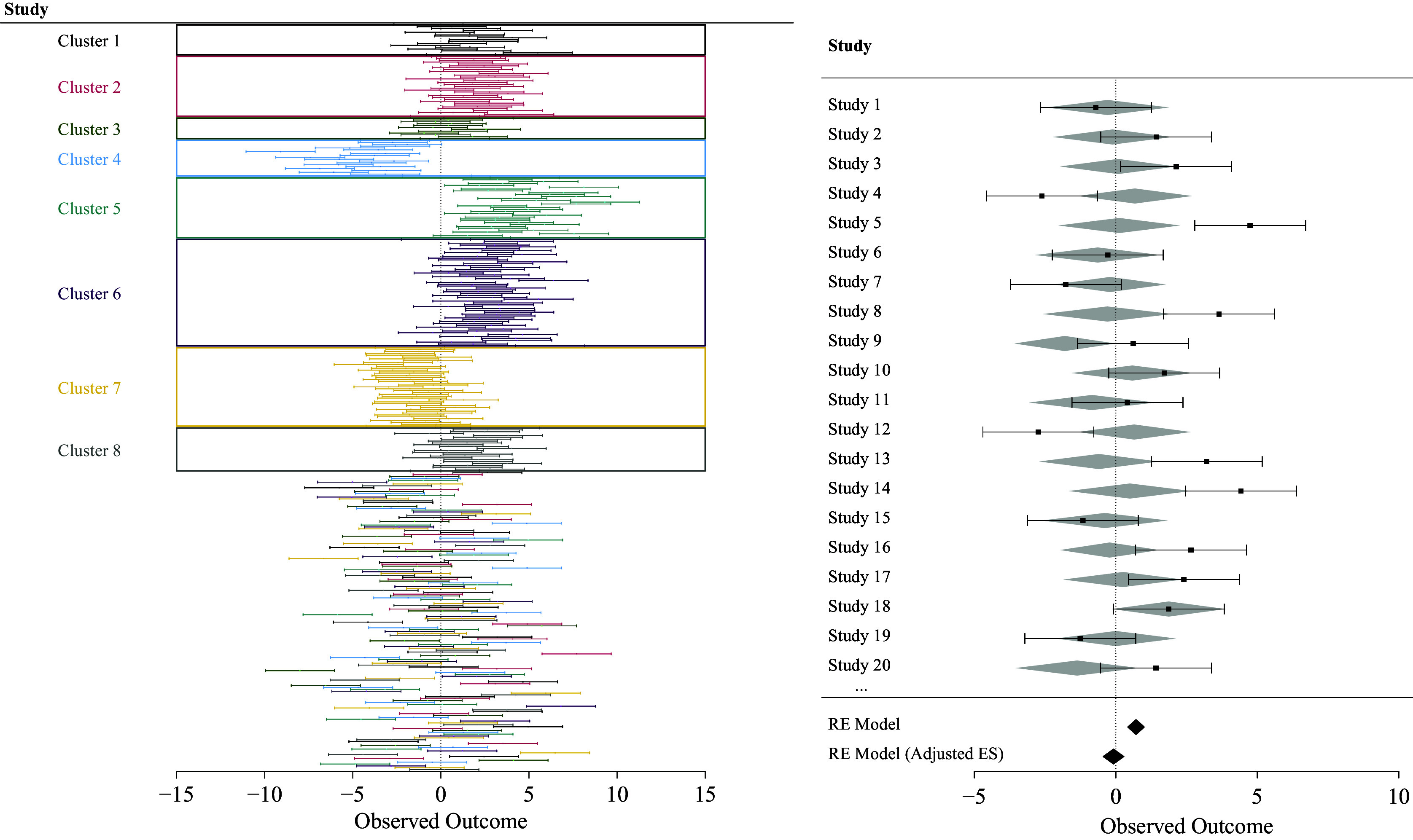
Forest plots of the simulated network analysed in Section 3.1. Top: Studies are ordered and coloured according to their cluster membership to identify deviant clusters. Bottom: Only 20 out of 400 total studies are shown. Grey-shaded diamonds signify effect sizes that were adjusted for the influence of paper proximity in the network. Black diamonds show model results of the models without and with (“adjusted effect”) authorship similarity matrix. Figure 3 Long description.

**Figure 4:**
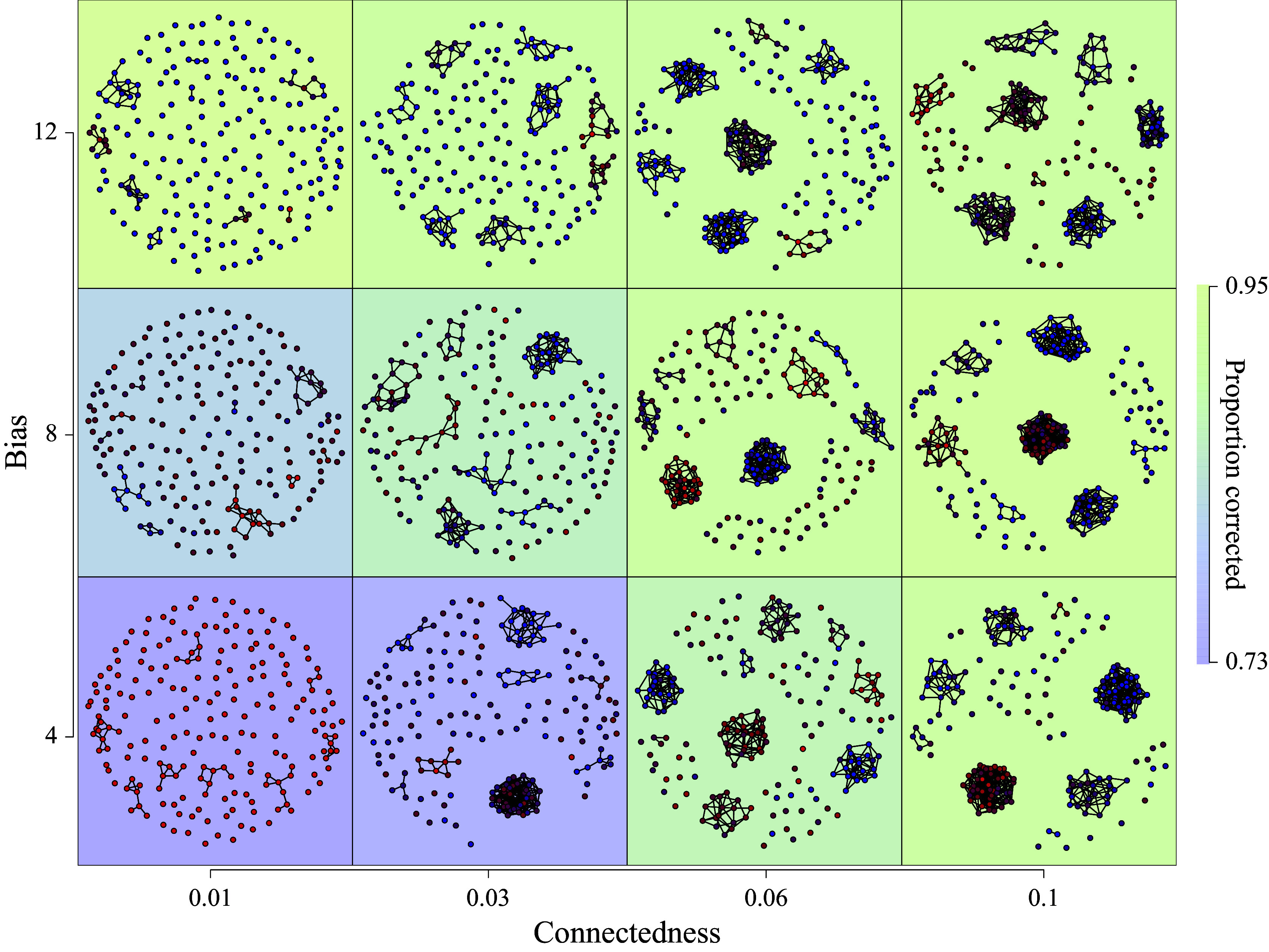
Influence of bias and connectedness of networks on the proportion of successfully corrected biased results. Proportions refer to corrected effect sizes out of 50 simulated networks for each combination. Proportions of success are the proportions of non-significant results for the full model, where the standard model resulted in a significant effect. Figure 4 Long description.

Because the correlogram relies on averaged effect size similarities for paper distance bins, it may, in some cases, miss small deviant clusters. To assess individual clusters, a forest plot with papers ordered and coloured by authorship cluster membership (Figure 3) can be helpful in identifying such deviants. Figure 3 shows such a forest plot. It can serve as a diagnostic tool as well as a typical visualisation of meta-analytical results showing the influence of the authorship bias at the level of a subset of individual studies, as well as the overall effect. Note that Figures 2 and 3 relate to the example values of Table 1. See Figure 4 for a more comprehensive overview of how the method performs over a wider range of scenarios.

As bias and connectedness increase, the method gets more reliable in correcting biased results (Figure 4). This explains the relatively low success rate at low bias–low connectedness panels, because it is less likely that the main assumption of the method is met. With low connectedness, there is less chance of bias propagation and significant effects are likely to be a result of random generation of data. If, by chance, such a network shows a significant effect, taking into account the authorship overlap is not expected to make a difference. With increasing connectedness both bias propagation and the possibility to correct for it increase as well. The latter is also true for larger bias. If a few clusters show large bias that lead to significant results, this large bias can be accounted for by the proximity of cluster members. For low bias networks, accounting for the proximity of cluster members does not make a difference, because all effect sizes are similar. So the success rate is linked to how likely authorship bias occurrence is for the depicted networks.

### 3.2 Published meta-analyses

Nine published meta-analyses were re-analysed to assess whether authorship network bias occurred and what its influence on the overall effect was. Four of them were used for the multilevel model approach of Moulin and Amaral (2020).^17^ The meta-analyses covered the scientific fields of psychology, biology, ecology, and medicine. They ranged from 10 to 457 primary studies. Data were taken from the respective meta-analyses or, where applicable, from the metafor package.

#### 3.2.1 Detection of authorship network bias.

**Figure 5:**
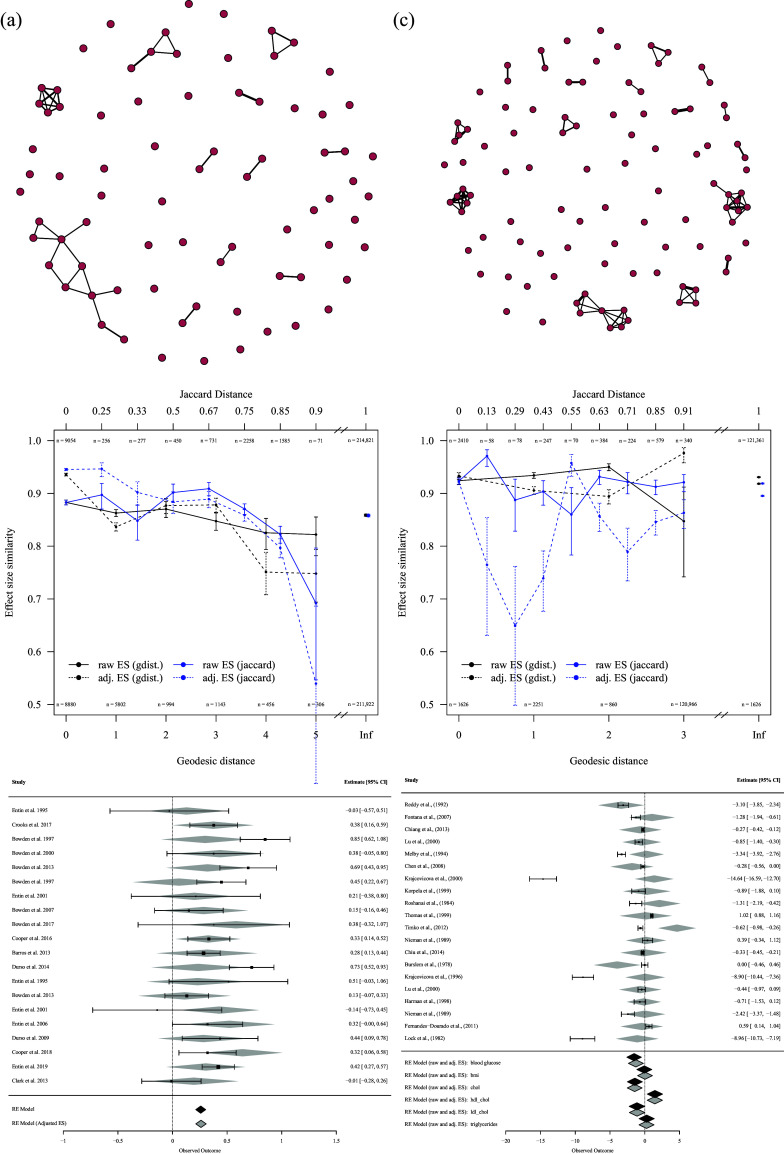
Diagnostic plots of (a) Bakdash et al. (2021),^30^ and (c) Dinu et al. (2017).^31^ See Appendix B of the Supplementary Material for other meta-analyses. Figure 5 Long description.

Most constructed networks showed substantial clustering (GCCs from 0.66 to 0.86) but extremely low overall connectedness (Table 2). The meta-analysis of Chen et al. (2014)^29^ shows a GCC of zero, because no closed triplets are present in the network. Figure 5 illustrates exemplary sets of diagnostic plots: the resulting networks, correlograms, and forest plots for Bakdash et al. (2021)^30^ and Dinu et al. (2017).^31^ Both networks show small and relatively evenly distributed clusters. In the correlograms, geodesic distance shows an effect on effect size similarity within clusters, albeit weak, and no effect at infinite distance. Jaccard distance seems to have no effect. Diagnostic plots for all analysed meta-analyses can be found in Appendix B of the Supplementary Material.

**Table 2:** Network characteristics and effect sizes before and after correction for the reanalysed meta-analyses Table 2 Long description.

Study	GCC	Connectedness	Raw effect	Adjusted effect	Bias
Bakdash et al. (2021)^30^	0.66	0.028	0.26	0.26	0.00
Besson et al. (2015)^32^	0.74	0.051	− 0.11	− 0.13	0.02
Dinu et al. (2017)^31^	0.89	0.019	− 1.53	− 1.28	0.25
			− 0.06	0.04	0.10
			− 1.43	− 1.45	0.02
			1.46	1.45	0.01
			− 1.13	− 1.11	0.02
			0.27	0.25	0.02
Gibson et al. (2011)^23^	0.83	0.035	0.47	0.43	0.04
Moura et al. (2021)^25^	0.86	0.004	0.37	0.37	0.00
Chen et al. (2014)^29^	0.00	0.030	− 0.65	− 0.65	0.00
Kredlow et al. (2016)^33^	0.75	0.133	0.60	0.70	0.10
Mathie et al. (2017)^34^	0.88	0.038	− 0.26	− 0.24	0.02
Munkholm et al. (2015)^35^	0.90	0.185	− 0.29	− 0.29	0.00

Taking into account the network metrics and the correlogram patterns, bias risk for the presented meta-analyses can be said to be low.

#### 3.2.2 Correction of authorship network bias.

Correction for the authorship network led to adjustment of effect sizes between 0 and 0.25 in the reanalysed studies (Table 2). In no case did the inclusion of the similarity matrix change the reported effect size enough to lead to a different conclusion (e.g., *p*-values remained on the same side of the significance thresholds). More important than a change in *p*-values, however, are changes in effect sizes.^2^ In our cases, those were also small for all but one moderator in Besson et al. (2015).^32^ The comparison of our proposed method to the multilevel approach used by Moulin and Amaral (2020)^17^ showed similar results for Chen et al. (2014),^29^ Kredlow et al. (2016),^33^ Mathie et al. (2017),^34^ and Munkholm et al. (2015).^35^

The resulting forest plots show some considerable differences in single effect sizes for a random sample of studies within the respective meta-analysis, but diamonds at the bottom show no difference in the overall estimate (a) or moderators (c).

## 4 Discussion

The main parameters determining the likelihood of encountering authorship network bias are the effect size differences of primary studies and the connectedness of the authorship network. Increases in both are required to lead to increased bias risk. The method presented effectively mitigates the bias when it occurs. Based on the meta-analyses included here, authorship network bias affecting the overall result of a meta-analysis is probably rare. However, authorship cluster affiliation has been shown to significantly impact reported results.^36^ With uneven authorship cluster sizes, this may lead to substantially biased overall results. For this reason and as there are no negative effects on the model’s estimates when the method is applied unnecessarily, it is recommended to apply it for all potentially affected authorship networks. The main cost is the time it takes to correct author names in the paper database, as not all authors are represented with unique names (such as researcher ID or ORCID). Incorrect naming could lead to missing edges where they should be included and to including edges where there should be none. The latter is of more concern, because it might down-weight the influence of whole clusters on the overall result, hence we analysed the effect of varying numbers of spurious edges, e.g., due to homonymous author names in different network scenarios. The effects of single or few spurious edges have proven to be negligible. See Appendix D of the Supplementary Material for the results of these simulations.

### 4.1 Similarity measures

For the quantification of overlap in authorship, there is no inherent reason to prefer Simpson, Sørensen, or Jaccard over other methods, such as node-correspondence methods^37^ or edit distances.^38^ When choosing a similarity measure, or when testing multiple, the resulting correlograms should be used to determine how well they capture the suspected bias in the network. This being said, we recommend using inverse geodesic distance as the standard similarity measure because of its ability to capture network structures exceptionally well. It may be complemented by adding other measures or even be replaced should the analyst find a better fitting alternative but it is a very good starting point in all cases.

For the simulated data, and by design, the model with geodesic similarity was clearly the better fit. In the re-analysed meta-analyses, neither geodesic distance, nor Jaccard, nor their combination explained much variance of effect sizes. Conceptually, geodesic similarity covers the idea of the authorship network bias better. It does seem sensible to also account for the proportion of shared authors, however. So, the heuristic combination of two measures seems a plausible way of analysing data. There are other ways of weighting network edges.^39^ ^–^ ^41^ However, for this set of meta-analyses, it seems that the effect of the edge weight does not carry much information. For larger effect size differences, there might be a larger effect and we would still recommend checking whether including weights changes the effect sizes at all.

The similarity matrix can be specified further. It seems plausible that not every author has the same influence on bias propagation. Depending on multiple aspects, authors’ ideas or suggestions might be taken more or less seriously by collaborators. Seniority and other character traits might influence this phenomenon but are somewhat difficult to quantify objectively. Other, more measurable things, such as academic rank or the respective H-index of the authors, could be included as a way of weighting edges in the network. Giving more weight to first, second, or last author (field specific) might also be an option. There are many ways to include other parameters in this method, which can be justified or refuted by assessing their effect on the results. Supplementary Material SC 7 includes a section on further weighting the included similarity matrices by taking into account the number of shared authors or some assigned measure such as H-index.

### 4.2 Influential parameters

For the range of connectedness values observed in our case studies, problematic levels of authorship network bias are unlikely. But even when effect size differences are small, with high connectedness (and high GCC), the results might still be changed considerably under authorship network bias. What a considerable change means is dependent on the respective outcome measure used. In any case, the importance of the similarity matrix can be assessed by calculating an $I^{2}$ equivalent for multilevel models, which quantifies how much of the total variance is explained by the similarity matrix.^42^ The bias risk decreases with diminishing effect size differences. Looking at GCC alone does not have much explanatory power for discovering problematic network structures. Only considering connectedness is slightly more helpful, but we recommend their joint interpretation to get an idea of the probability that we encounter authorship network bias.

No single diagnostic measure is sufficient for checking bias patterns. The correlogram is useful but only reflects mean effect size similarity for each distance category, potentially missing outlier clusters. With enough data, this is less problematic, but relying solely on it risks underestimating bias. Another approach is to colour network nodes by reported effect sizes (as in Figure 1) or to colour studies and effect sizes according to authorship cluster membership in a forest plot (see Figure 3 and Supplementary Material SC 2 and SC 7 for code) and, most reliably, to compute the similarity matrix for inclusion in the model.

### 4.3 Limitations and extensions

How informative the authorship network is, depends on which primary studies are included. This means the method can never fully capture all possible bias leakage, for example, from connections outside the depicted networks, but also due to mentoring, supervision, and funding review. Moulin and Amaral (2020)^17^ proposed an extension to their method, also applicable here: with unique author identifiers and lifetime collaboration networks, collaborations beyond the meta-analysis could be incorporated.

Our method can reliably detect and correct bias in high-bias–high-connectedness networks with diminishing success rate as both decrease. It performs worst with little effect size variance within sparse networks. In these cases, the potential for influential spurious edges is also highest. They have, however, been shown to have little to no effect on simulated networks within a realistic range of wrongfully added connections.

### 4.4 (Dis)advantages relative to other methods

The issue of non-independence of effect sizes or studies and its adverse influence on meta-analyses is not new. Initially, concerns focused on nested effects and hierarchical data structures.^11^ ^,^ ^22^ The former study highlighted that hierarchy can also arise purely from meta-analytical data, where effect sizes nest within studies, which in turn nest within researchers or labs. Moulin and Amaral (2020)^17^ extended this by adding another clustering level to account for authorship networks.

A different approach was chosen here, due to key advantages of modelling a *network* over a multilevel structure. Using a similarity matrix instead of assigning clusters better captures relationships within clusters—more distant papers within clusters might exhibit less effect size similarity, a pattern lost in standard multilevel models. Nestedness is also not always clear-cut, whereas a similarity matrix works regardless of cluster structure. Additionally, this method imposes no cluster size restrictions; even two-paper clusters can add explanatory power.

Multilevel models, by contrast, are recommended to have sample sizes of at least 20 data points per cluster per level.^43^ ^,^ ^44^ These recommendations would often be ignored, which may lead to substantially biased estimates^43^ ^,^ ^45^ or clustering would be ignored altogether. Bell et al. (2008)^44^ further show that a high number of isolates inflates confidence intervals and increases type II error risk—an issue exacerbated in authorship networks due to even higher proportions of isolates. With a similarity matrix, confidence intervals only expand when the overall effect changes, avoiding this issue.

In comparison with Moulin and Amaral (2020),^17^ visual assessment of networks is more directly linked to potential bias propagation, when vertices represent papers instead of authors. In the latter case, strong clustering might just be a result of one or few papers being authored by many people, not requiring any overlap.

Using both methods on the same published meta-analyses showed no relevant differences in the reported results. Differences are not to be expected in cases with large clusters, high intra-cluster connectivity, and clear separation between clusters. The latter two seem to be commonly encountered in real meta-analyses. For networks with potentially problematic properties for multilevel models, such as small and gradually connected rather than clearly separated clusters, our proposed method may prove advantageous. When there is no clear indication for the use of one of the methods, they may both be applied and results compared.

### 4.5 When to use this method

Given the relatively minor changes in effect sizes observed in the re-analysed meta-analyses, the proposed method may be perceived as addressing a largely hypothetical concern. Authorship network bias appears to be small and rare; however, it is important to acknowledge that only nine meta-analyses were examined. While its impact on effect sizes seems minimal, the underlying concept of bias propagation remains a plausible concern in real-world meta-analyses. This, along with the potential influence of authorship cluster membership studies’ results,^36^ provides sufficient justification for considering this method as a standard measure. Although such bias may not significantly affect most cases’ overall results, every measure to reduce the likelihood of Type I errors should be considered, particularly when it does not increase the risk of Type II errors. Therefore, when a meta-analysis network exhibits authorship overlap, incorporating the authorship network provides a straightforward approach to mitigating the risk of biased results.

This method gains in importance when dealing with clustered networks, in which single or few author clusters account for large proportions of primary studies (e.g., right panels in Figure 1). Assessment of a coloured forest plot (Figure 3) is a helpful tool to assess the potential impact these clusters may have on the result of the meta-analysis and can help in deciding whether correction is needed. However, because of the above points, we recommend routinely implementing this method (or other ways of mitigating authorship bias), even when diagnostic measures indicate low bias risk. Ignoring authorship network bias may lead (and has led) to obscured overall effects due to a single deviant cluster.^46^

### 4.6 Implementation

We present a user-friendly approach for identifying and addressing authorship network bias, outlining the fundamental concepts, assumptions, and comprehensive example analyses. Since all steps were performed using existing functions in the R environment, researchers can readily implement our method in their own meta-analyses. It is seamlessly integrated into the analyst’s workflow and does not require any additional change in model structure. It may be used in basic random effects models, hierarchical dependence models, correlated effects models, and the combination thereof,^47^ as long as no other approach of dealing with authorship network bias has been implemented. The only modification lies in providing an additional random effect term and a corresponding similarity matrix. A detailed tutorial, along with the complete code for all studies and simulations referenced in this work, is provided in Appendix E of the Supplementary Material.

### 4.7 Conclusion

Meta-analysis is generally seen as a reliable method to come to broader conclusions and generalise knowledge better than any single study could. As such, meta-analysis is often trusted more than each single primary study included, because of a broader perspective on topics, larger sample sizes, and higher statistical power.^48^ This trust is only justified if meta-analyses are conducted avoiding the many potential pitfalls of that method. Criticism has been voiced about the general idea of meta-analysis,^4^ the inevitable complexity, which cannot be steered through without making mistakes,^49^ and its wrong use and interpretation of its results.^4^ While Borenstein et al.^2^ agree with these points and highlight the problems that arise from a misuse of meta-analytical methods, they also argue that all of them can be countered with increasing education on proper implementation of methods. One such problem is the introduction of biases specific to meta-analysis. The present work helps in bringing a potentially influential bias to the reader’s attention and proposes a way to account for it. This work, by drawing further attention to the authorship network bias, along with a diagnostic tool and a method to remedy it, aims at further improving the quality of meta-analysis and reducing the likelihood of reporting biased results.

## Supporting information

10.1017/rsm.2025.10063.sm001 Rieck et al. supplementary material Rieck et al. supplementary material

## Data Availability

Data and Code are available at https://doi.org/10.5281/zenodo.17712269.

## Supplementary material

The supplementary material for this article can be found at https://doi.org/10.1017/rsm.2025.10063.

## Long descriptions

### Figure 1 Long description

A network diagram features a central cluster of nodes surrounded by seven distinct peripheral clusters and numerous isolated nodes. Nodes are colored on a gradient from red to blue representing effect sizes. At the center is a dense, circular cluster of dark purple nodes. Moving outward, clusters of varying densities are distributed in a circular pattern. Some clusters are tightly interconnected with black lines, while others are more sparse. Yellow nodes within some clusters indicate the bias origin.

To the left of the network is a list of Input values

No. of papers 400

No. of clusters 8

No. of isolates 160

Cluster density 0.3

Cluster evenness 0.9

Bias origin random

Bias type individual

Homophily 0.7

Isolates s d 3

Cluster s d 1

To the right of the network is a list of Output values

161 No. of isolates

0.28 Cluster density

0.95 Cluster evenness

2.96 Isolates s d

1.49 Cluster s d

At the bottom, a legend for Effect sizes shows a color scale from red for negative 9, through purple for 1, to blue for 9. A yellow square indicates Bias origin.

Navigate back to Figure 1.

### Figure 2 Long description

Two panels show E S similarity on the y-axis ranging from 0.70 to 1.00.

Left Panel: Geodesic Distance Model.

* The x-axis shows geodesic distance from 1 to 6, followed by a break and Inf.

* Raw E S is a solid black line with circular markers starting at 0.91 for distance 1 and gradually decreasing to 0.84 at distance 6.

* Adjusted E S is a dashed gray line with diamond markers starting at 0.83, fluctuating between 0.78 and 0.85, and ending at 0.77 at distance 6.

* Vertical error bars represent confidence intervals, which widen significantly as distance increases. At distance 5, the error bar extends from 0.70 to 1.00.

* Sample sizes n are listed at the bottom, decreasing from 1259 at distance 1 to 2 at distance 6, with 75,539 at Inf.

Right Panel: Jaccard Model.

* The x-axis shows Jaccard similarity values: 0.29, 0.26, 0.23, 0.19, 0.16, 0.13, followed by a break and 0.

* Raw E S is a solid black line with circular markers remaining relatively stable between 0.91 and 0.94.

* Adjusted E S is a dashed black line with circular markers fluctuating between 0.83 and 0.89.

* Confidence intervals widen as Jaccard similarity decreases toward 0.13.

* Sample sizes n range from 279 at 0.29 similarity to 5 at 0.13 similarity, with 78,541 at 0.

Navigate back to Figure 2.

### Table 1 Long description

The table consists of five columns: Parameter, Explanation, Example value, Simulation range, and Observation range.

* No. of papers: For the simulation, this is the same as the number of effect sizes. Example: 400. Simulation range: 20 to 1,000. Observation range: 10 to 457.

* No. of clusters: Number of disjunct authorship clusters. Example: 8. Simulation range: 1 to 20. Observation range: 2 to 52.

* No. of isolates: Number of non-connected papers. Example: 160. Simulation range: 20% to 80%. Observation range: 40% to 60%.

* Cluster density: Proportion of all possible connections in a cluster or likelihood of any two cluster members to be directly connected. Example: 0.3. Simulation range: 0.2 to 1. Observation range: 0.79 to 0.95.

* Cluster size evenness: Shannon Evenness of clusters. Example: 0.9. Simulation range: 0.5 to 1. Observation range: 0.54 to 1.

* Bias size: Standard deviation of normal distribution from which bias origin values are drawn. Example: 5. Simulation and Observation ranges are not applicable.

* sigma sub cluster: Standard deviation of normal distribution around bias origin from which rest of cluster values are drawn. Example: 1.

* Bias origin: One of: most central, marginal, or random cluster member. Example: random.

* Bias type: Either whole cluster has same bias (cluster) or decreasing influence of bias origin with increasing geodesic distance (individual). Example: individual.

* Homophily: Sets influence of bias origin on rest of cluster (0 to 1). Example: 0.7.

* sigma sub isolates: S D of normal distribution from which values of non-connected papers are drawn. Example: 3.

Navigate back to Table 1.

### Figure 3 Long description

A two-panel forest plot visualization.

Top-left panel: A large-scale forest plot with the y-axis labeled Study and the x-axis labeled Observed Outcome ranging from minus 15 to 15. Data points are grouped into eight colored rectangular boxes representing clusters.

- Cluster 1: Grey points centered near 0.

- Cluster 2: Pink points shifted slightly positive.

- Cluster 3: Green points centered near 0.

- Cluster 4: Light blue points shifted negative, between minus 5 and minus 10.

- Cluster 5: Teal points shifted positive, between 0 and 5.

- Cluster 6: Purple points centered near 2.

- Cluster 7: Yellow points shifted negative, between 0 and minus 5.

- Cluster 8: Grey points centered near 0.

Below these boxes, a dense mix of individual study lines from all clusters is shown without grouping.

Bottom-right panel: A detailed forest plot showing 20 individual studies. The x-axis ranges from minus 5 to 10. Each study row contains:

- A black square representing the observed effect size with horizontal error bars for confidence intervals.

- A grey-shaded diamond representing the adjusted effect size for that study.

At the bottom of this panel, two summary diamonds are shown:

- R E Model: A black diamond centered slightly above 0.

- R E Model open parenthesis Adjusted E S close parenthesis: A black diamond centered slightly below 0, indicating the influence of the authorship similarity matrix.

Navigate back to Figure 3.

### Figure 4 Long description

A multi-panel grid with three rows and four columns. The vertical y axis is labeled Bias with tick marks at 4, 8, and 12. The horizontal x axis is labeled Connectedness with tick marks at 0.01, 0.03, 0.06, and 0.1.

Each of the twelve panels contains a circular arrangement of nodes and clusters.

* Moving from left to right along the Connectedness axis, the networks transition from mostly isolated nodes to increasingly dense and interconnected clusters.

* Moving from bottom to top along the Bias axis, the background color of the panels shifts from light purple to light green.

A color scale legend on the far right is labeled Proportion corrected. It ranges from a light purple at the bottom representing 0.73 to a light green at the top representing 0.95.

In the bottom-left panels (low Connectedness and low Bias), the nodes are predominantly red and isolated. In the top-right panels (high Connectedness and high Bias), the nodes form tight, dark blue and purple clusters. The density of connections within the clusters increases significantly as Connectedness values move from 0.01 to 0.1.

Navigate back to Figure 4.

### Table 2 Long description

The table consists of six columns: Study, G C C, Connectedness, Raw effect, Adjusted effect, and Bias.

* Bakdash et al. 2021: G C C 0.66, Connectedness 0.028, Raw effect 0.26, Adjusted effect 0.26, Bias 0.00.

* Besson et al. 2015: G C C 0.74, Connectedness 0.051, Raw effect minus 0.11, Adjusted effect minus 0.13, Bias 0.02.

* Dinu et al. 2017: G C C 0.89, Connectedness 0.019. This study includes six sub-rows of effect data. The first row shows Raw effect minus 1.53, Adjusted effect minus 1.28, Bias 0.25. Subsequent rows show Raw effects of minus 0.06, minus 1.43, 1.46, minus 1.13, and 0.27, with corresponding Adjusted effects and Bias values ranging from 0.01 to 0.10.

* Gibson et al. 2011: G C C 0.83, Connectedness 0.035, Raw effect 0.47, Adjusted effect 0.43, Bias 0.04.

* Moura et al. 2021: G C C 0.86, Connectedness 0.004, Raw effect 0.37, Adjusted effect 0.37, Bias 0.00.

* Chen et al. 2014: G C C 0.00, Connectedness 0.030, Raw effect minus 0.65, Adjusted effect minus 0.65, Bias 0.00.

* Kredlow et al. 2016: G C C 0.75, Connectedness 0.133, Raw effect 0.60, Adjusted effect 0.70, Bias 0.10.

* Mathie et al. 2017: G C C 0.88, Connectedness 0.038, Raw effect minus 0.26, Adjusted effect minus 0.24, Bias 0.02.

* Munkholm et al. 2015: G C C 0.90, Connectedness 0.185, Raw effect minus 0.29, Adjusted effect minus 0.29, Bias 0.00.

Navigate back to Table 2.

### Figure 5 Long description

The figure consists of two columns. The left column represents Bakdash et al. and the right column represents Dinu et al.

Top Row: Network diagrams showing nodes as red circles. In panel a, nodes are mostly isolated with a few small clusters of two to five nodes. In panel c, nodes are more densely clustered with several complex interconnected groups.

Middle Row: Line graphs plotting Effect size similarity on the y axis from 0.5 to 1.0. The bottom x axis is Geodesic distance from 0 to 5 and Infinity. The top x axis is Jaccard Distance from 0 to 1. Four lines are shown: raw E S g dist in solid black, adj. E S g dist in dashed black, raw E S jaccard in solid blue, and adj. E S jaccard in dashed blue. In both panels, similarity generally decreases as distance increases, with the blue jaccard lines showing a sharper decline than the black geodesic lines.

Bottom Row: Forest plots listing individual studies on the left and their estimates with 95 percent C I on the right. Panel a shows approximately 20 studies with points and horizontal error bars centered around an observed outcome of 0.5. Panel c shows a larger set of studies with error bars mostly clustered around 0, though some outliers extend far to the left. Both plots conclude with diamond shapes representing the R E Model and R E Model Adjusted E S at the bottom.

Navigate back to Figure 5.
