# Supplementary material for: Authorship network bias in meta-analysis
Source: Res Synth Methods. 2026 Feb 18;17(4):734–49. doi: 10.1017/rsm.2025.10063 (PMC13311356; doi:10.1017/rsm.2025.10063)
Supplement: Rieck et al. supplementary material [file S175928792510063Xsup001.pdf]

Appendices to Authorship network bias in meta-analysis

A Simulation structure

Data were simulated with the following structure:

1. Create data frame with n_{paper} entries
2. Assign $n_{paper} - n_{isolates}$ into one of $n_{cluster}$. How many papers per cluster is set by $cluster\ size\ evenness$
3. Create overlap matrix with $cluster\ density$ chance of pairwise connections per cluster. Entries are 0 and 1 for non-connected or connected respectively. Note that this means it is possible for a cluster to turn out as only isolates. This is more likely to happen with low $cluster\ density$ values and small cluster sizes (because of uneven cluster size distribution or overall low n_{paper}). In that case the simulation was run again.
4. Create network and extract geodesic distance matrix.
5. Select one of the network nodes as $bias\ origin$. Selection could be *central* (node with highest degree or most edges connecting to it), *marginal* (node with lowest degree) or *random*.
6. Assign strength of bias as $bias\ origin$ from $N(0, bias\ size)$
7. Assignment remaining studies' effect sizes from

$$N(\mathit{effect}_{bias\ origin} * homophily * \frac{1}{geodesic\ distance\ to\ bias\ origin}, \sigma_{cluster})$$

8. Assignment isolates' effect sizes from $N(0, \sigma_{isolates})$

B Additional plots

We provide the respective authorship networks, correlograms (pre and post correction), and forest plots with raw and adjusted effect sizes for:

(a) Bakdash et al. 2021[1]
(b) Besson et al. 2015[2]
(c) Dinu et al. 2017[3]
(d) Gibson et al. 2011[4]
(e) Moura et al. 2021[5]
(f) Chen et al. 2015[6]
(g) Kredlow et al. 2016[7]
(h) Mathie et al. 2017[8]
(i) Munkholm et al. 2015[9]

The forest plots show a subset of 20 effect sizes per meta-analyses, unless it consisted of fewer.

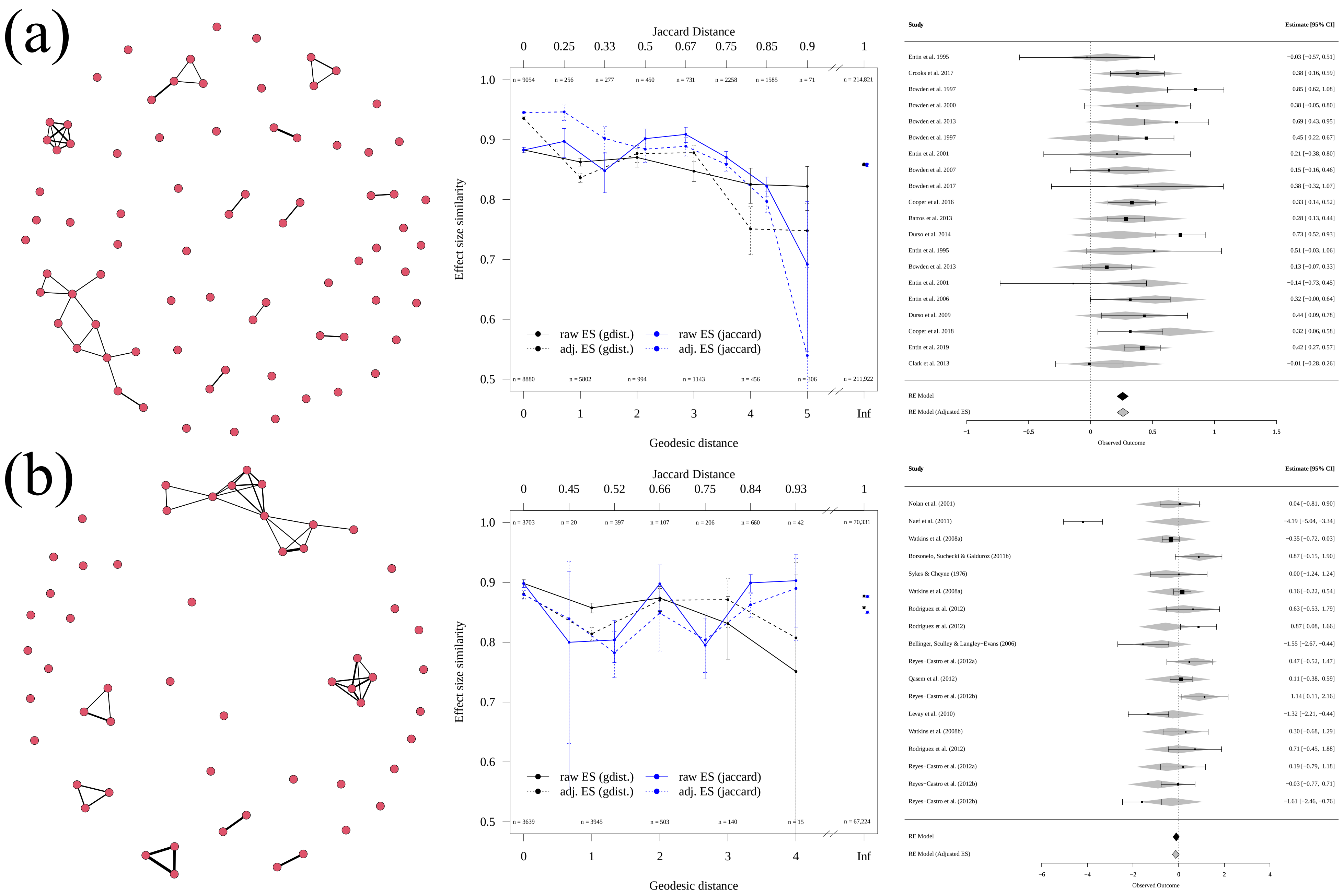

Figure 1: authorship overlap networks, correlograms, and forest plots for meta-analyses in section 3.2

.

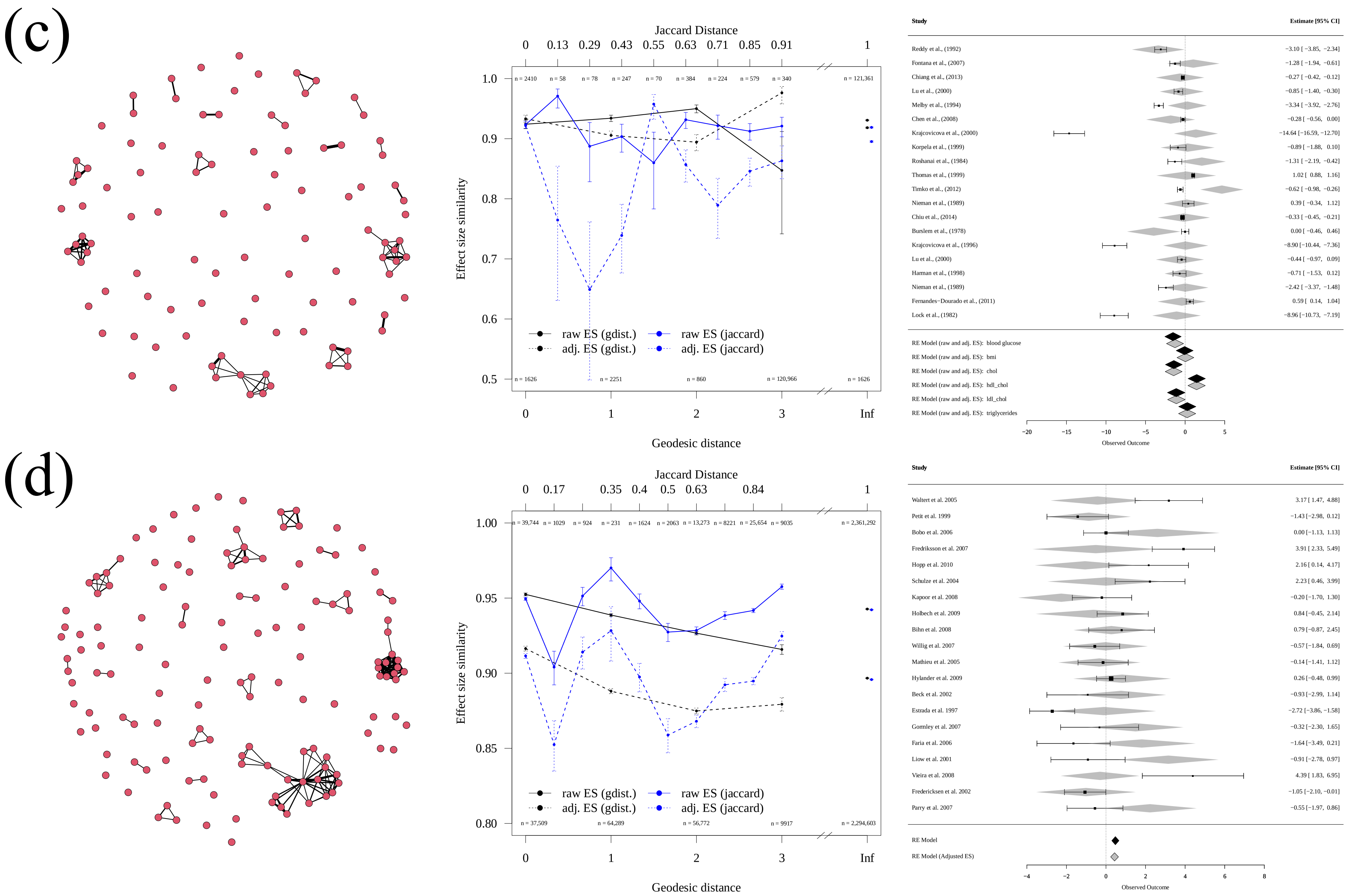

Figure 2: authorship overlap networks, correlograms, and forest plots for meta-analyses in section 3.2.

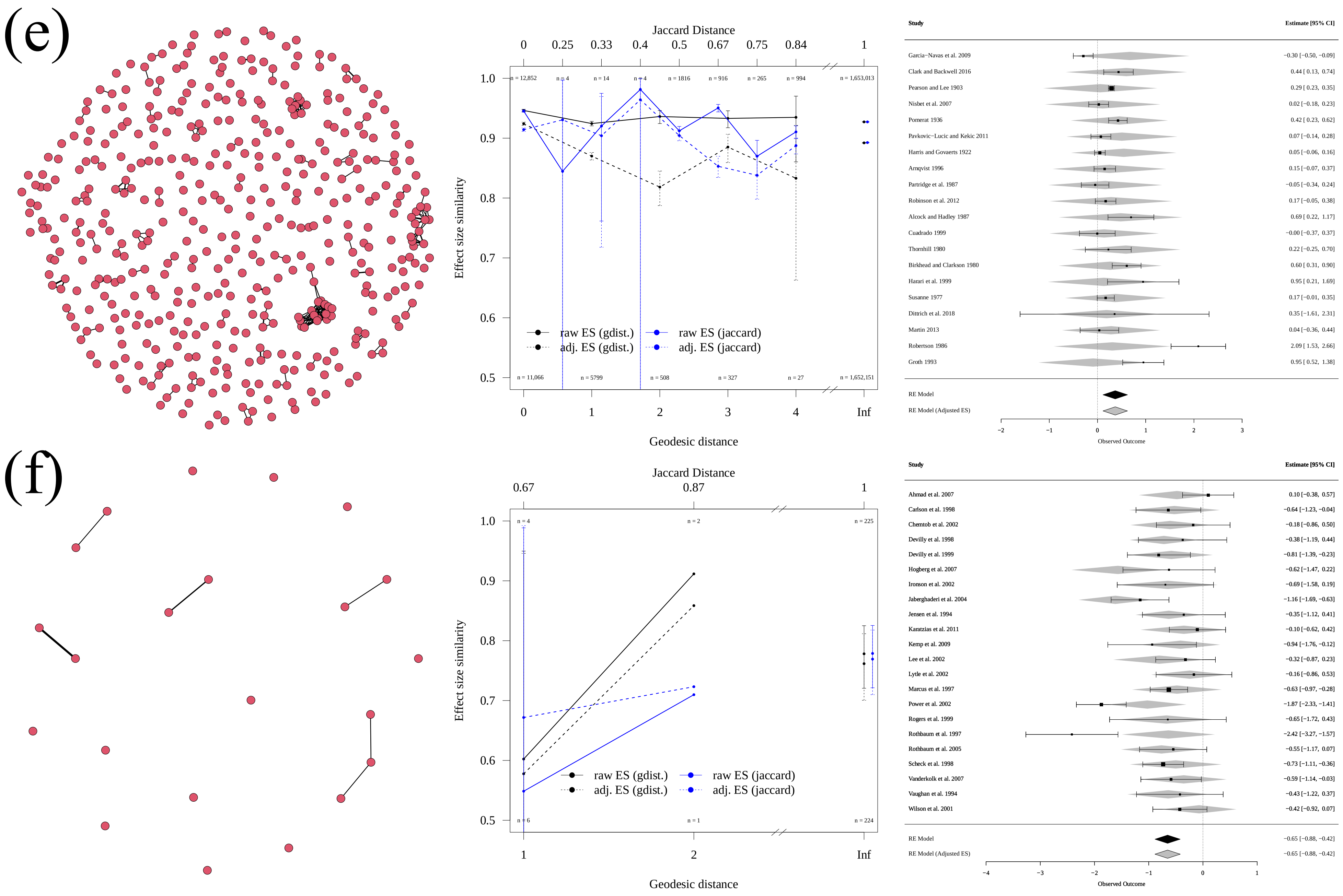

Figure 3: authorship overlap networks, correlograms, and forest plots for meta-analyses in section 3.2.

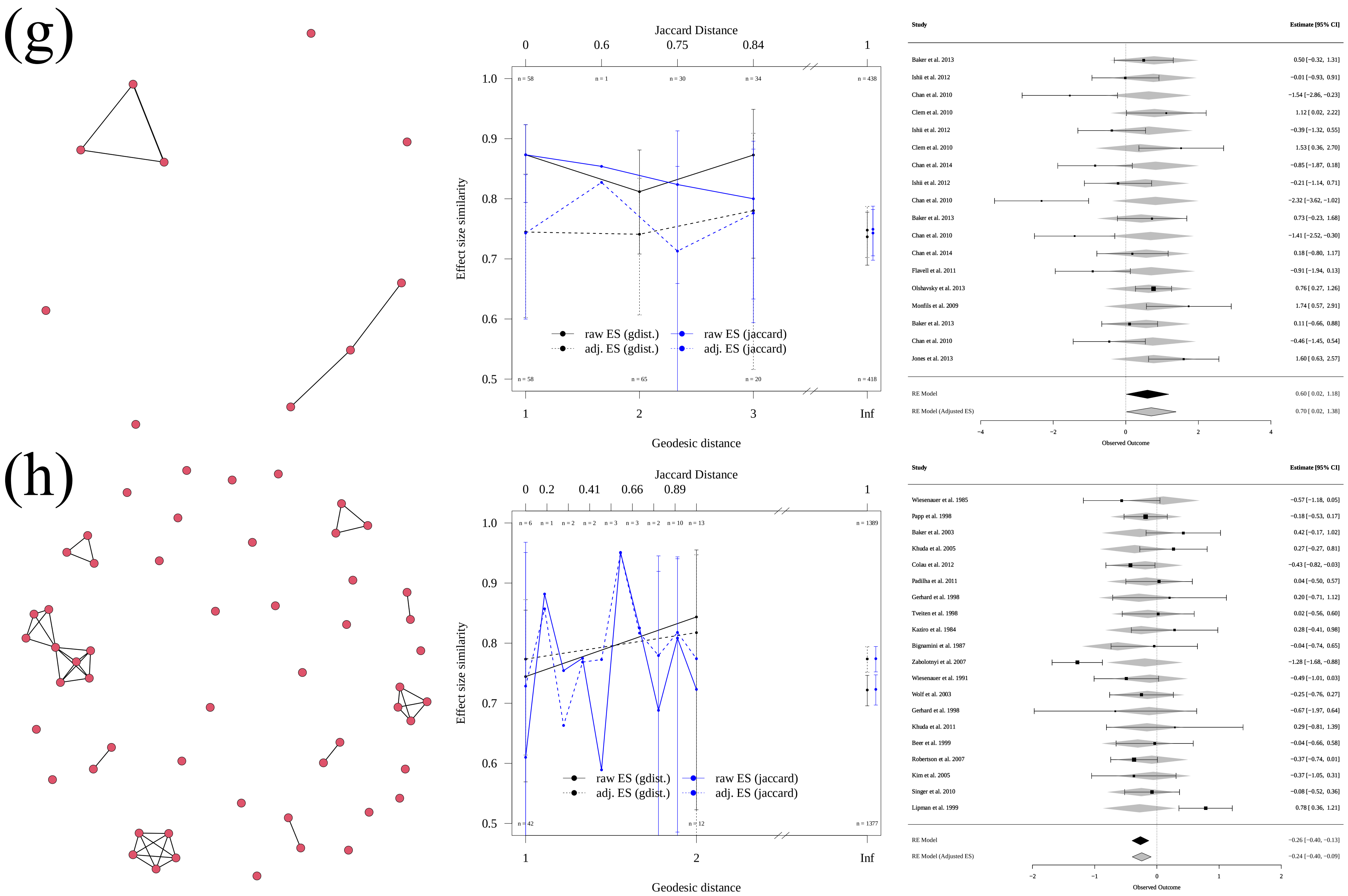

Figure 4: authorship overlap networks, correlograms, and forest plots for meta-analyses in section 3.2.

(i)

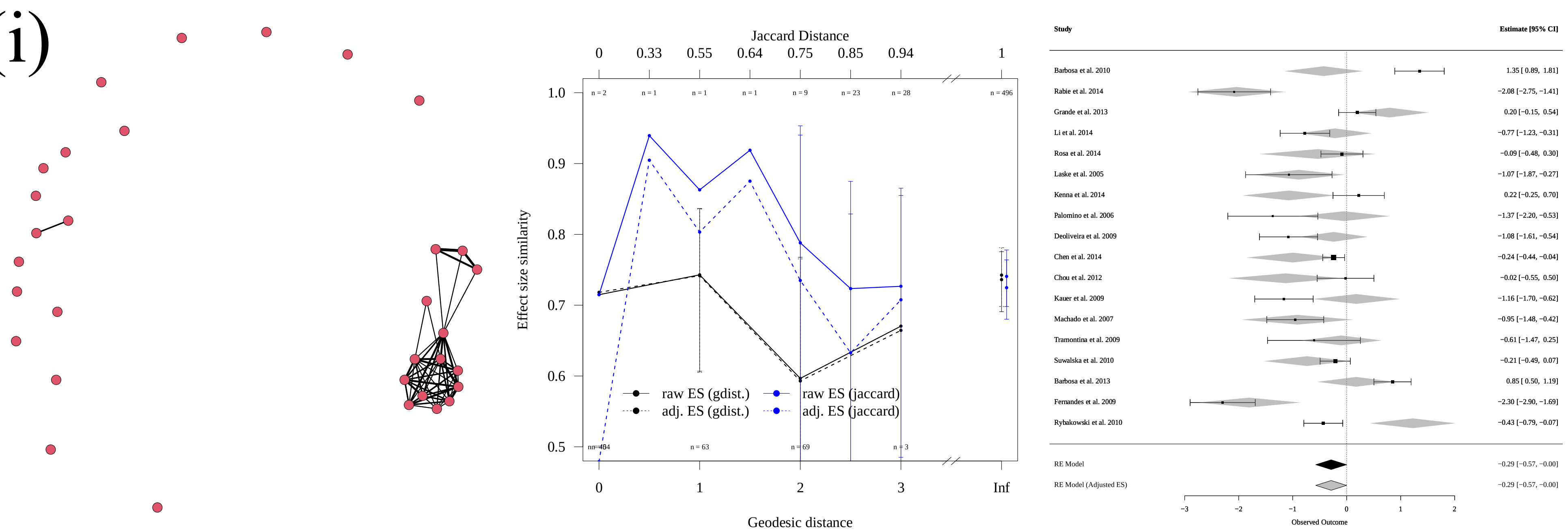

Figure 5: authorship overlap networks, correlograms, and forest plots for meta-analyses in section 3.2.

C How to: Reanalysis of Gibson et al. (2011)

The original study analysed not only the overall mean effect of disturbances on tropical biodiversity (as done here), but also included moderators in the model. To keep the proportion of code minimal, this will not be replicated here. Gibson et al. (2011)[4] also manually changed some of the values' signs, as for some taxa they expected biodiversity to be higher in the disturbed areas as a sign of said disturbance (e.g. seedlings, early successional species, open land species, etc.). This approach was not replicated here. The overall effect is, apart from the described difference, the same as the reported overall effect. All analyses were conducted with R version 4.3.1[10].

C.1 Packages used in this example

```
library(vegan)
library(dplyr)
library(statnet)
library(stringr)
library(metafor)
library(ncf)
library(igraph)
```

C.2 Data preparation

By the end of this first section we should have a `data.frame` with either the effect sizes and sampling variances or the required information to calculate them (e.g. with metafor's `escalc` function). Our data frame should include a study identifier column, numbered observations (if there are multiple effect sizes per study) and a column with the respective author names of the included studies (as a list of vectors) (see Table 1). I would recommend removing all special characters and non-regular expressions from all author names. This is a helpful first step but does not replace going through the author list manually to check for typing errors, in- or excluded second names and so forth.

We are largely going to skip this section, because it is very hard to generalise, but here are some potentially useful lines of code:

```
strsplit(x, "\\,|\\&") # splits chr of all authors into separate chrs
iconv(x, from = 'UTF-8', to = 'ASCII//TRANSLIT') # removes accents
gsub("WallaceAR", "WallaceA", x) # removes middle names etc.
```

For the complete code (including data preparation), see supplementary material SC 5.

Table 1: Part of exemplary data frame for further analysis.

Study ID	Observation	Effect size	Sampling Variance	Authors	[...]
1	1	1.32	0.49	c("BarlowJ.", "GardnerT.A.",[...]	[...]
1	2	0.56	0.42	c("BarlowJ.", "GardnerT.A.",[...]	[...]
2	1	3.82	1.44	LammertinkM.	[...]
3	[...]	[...]	[...]	[...]	[...]

C.3 Creation of authorship network

Much of this code was taken and slightly modified from Carlen (2019)[16].

Here we create a matrix with studies as columns, authors as rows and true or false as entries to indicate who authored what.

```
X_author_list <- lapply(X$authors, function(x){strsplit(x, "\\,|\\&")})
X_author_list <- sort(unique(unlist(X_author_list)))
X_studies <- X[!duplicated(X$study.ID), ]
X_bipartite_edges <- lapply(X_studies$authors, function(x){X_author_list %in% x})
X_bipartite_edges <- do.call("cbind", X_bipartite_edges)
rownames(X_bipartite_edges) <- X_author_list
```

From this we create a matrix with study ID as both rows and columns and the number of shared authors as entries.

```
X_shared_authors <- t(X_bipartite_edges) %*% X_bipartite_edges
```

This matrix is then used to display the authorship overlap network.

```
X_network <- as.network(X_shared_authors, directed = F)
plot.network(X_network, edge.col = "black",
edge.lwd = X_shared_authors)
```

The network plot is shown in Figure 2 (d).

Next, get geodesic distance matrix:

```
X_geo.dist <- geodist(X_network)
```

C.4 Calculation of effect size similarities

Get effect size distances:

```
X_effect_dist_mat <- as.matrix(dist(X_effect$yi))
```

Convert to similarities:

```
X_effect_sim_mat <- 1 - (X_effect_dist_mat/max(X_effect_dist_mat))
```

From the 138 x 138 (number of studies) geodesic distance matrix, calculate 2220 x 2220 (number of effect sizes) matrix:

```
X_geo_dist_mat <- X_geo.dist$gdist
colnames(X_geo_dist_mat) <- 1:ncol(X_geo_dist_mat)
effects_per_study <- X_effect %>% group_by(study.ID) %>% count()
geo_dist_vec <- vector()
for(i in 1:138){
  geo_dist_vec <- c(geo_dist_vec, rep(rep(X_geo_dist_mat[i, ],
  times = effects_per_study$n),
  times = effects_per_study$n[i]))
}
X_geo_dist_mat_full <- matrix(data = geo_dist_vec, ncol = 2220, nrow = 2220)
colnames(X_geo_dist_mat_full) <- 1:ncol(X_geo_dist_mat_full)
```

Then create the correlogram:

```
X_mantel_corlog <- ncf::mantel.correlog(X_geo_dist_mat_full, X_effect_sim_mat,
    increment = 1, resamp = 1)
tail(X_mantel_corlog$correlation, n = 1) # returns cor at geodist = Inf
```

We can inspect the values of the correlogram or plot it. If we plot it, we should include the similarity at infinite geodesic distance:

```
plot(X_mantel_corlog$correlation)
```

The resulting correlogram is shown in Figure 2 (d).

C.5 Calculation of distance metrics

From the previously determined similarity at infinite geodesic distance (0.94), we are going to calculate our inverse geodesic distance matrix. We have to manually change the -Inf values to 0. The column names need to correspond to the study ID in the model later. So for cases where some rows were dropped from the model due to missing values, we need to assign only the non-missing values' study IDs here. Another option would be to exclude missing values before fitting the model.

```
X_inv.geo <- 1 - tail(X_mantel_corlog$correlation, n = 1) * X_geo.dist$gdist / max(X
    _geo.dist$gdist[which(is.finite(X_geo.dist$gdist))])
X_inv.geo[X_inv.geo == -Inf] <- 0
```

```
colnames(X_inv.geo) <- 1:ncol(X_inv.geo)
```

In the code in supplementary material SC 5, readers find the same procedure, along with a function to calculate Jaccard values and the code to combine the two similarity matrices. Because the procedure is exactly the same, just with different values, here I included only the inverse geodesic distance option.

C.6 Analysis

Before we can run our models, we need to do some more preparation: Metafor's rma.mv function allows us to include similarity matrices (for things such as phylogenetic meta-analyses). Because we cannot include the same column in the random part of the model twice, we first create a new column to which we will later attach our *phylogeny*:

```
X_effect$study.ID.phyl <- X_effect$study.ID
```

These next few lines are not necessary for the analysis itself, but for visualisations such as forest or funnel plots the cluster membership might be useful to detect problematic trends within the clusters.

```
X_effect$membership <- NA
walktrap <- graph_from_adjacency_matrix(X_shared_authors) |> cluster_walktrap()
counts <- table(X_effect$study.ID)
X_effect$membership <- as.factor(rep(walktrap$membership, times = counts))
```

Now that our data are ready for the analysis, we start with the simple model, without including the similarity matrix. We can of course skip this step and go straight for our final model, but seeing how much the result differs might be of interest.

```
X.rma.null <- rma.mv(yi, V = vi, random = list(~1|study.ID/obs), data = X_effect)
```

With the summary function we could now get our overall effect size. But we will first run an exemplary model with one of our similarity matrices.

```
X.rma.full.invgeo <- rma.mv(yi, V = vi, random = list(~1|study.ID/obs, ~1|study.ID.
    phyl), R = list(study.ID.phyl = X_inv.geo), data = X_effect)
```

We can do this for all of our constructed similarity matrices and then compare the results (Figure 6).

C.7 Model fit

To choose the best fitting model and to check whether our model with the similarity matrix was able to handle the undesired correlation, we need to look at adjusted effect sizes: First, get best linear unbiased predictions (BLUP) for all random effects:

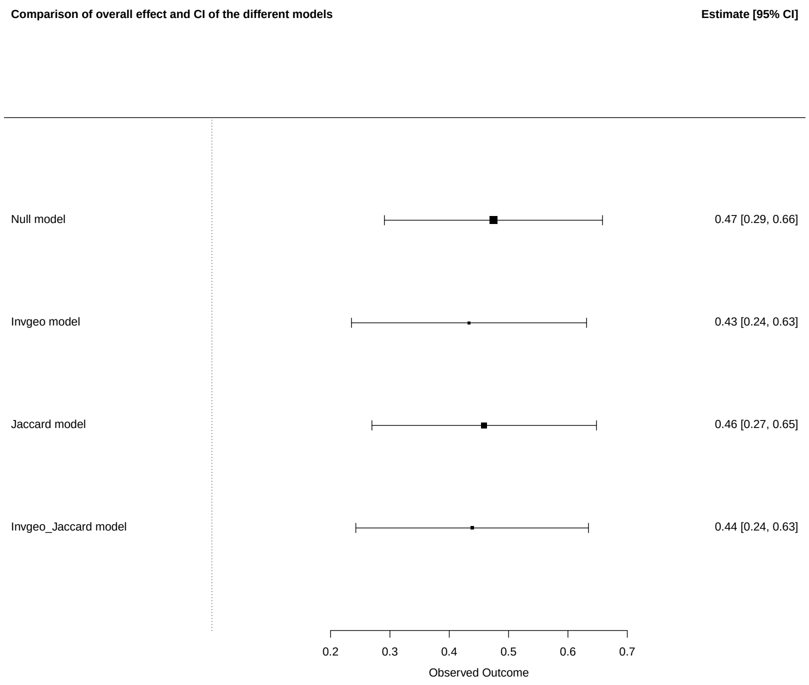

Figure 6: Model comparison for Gibson et al. (2011)[4].

```
X_ranef_invgeo <- ranef(X.rma.full.invgeo)
```

Because in this case not all random effects have the same length, we need to repeat the BLUP for study ID as many times as there are effects reported in that study:

```
rep_vec <- X_effect %>% group_by(study.ID) %>% count()
```

With that we can calculate the adjusted effects:

```
adjusted_effects_invgeo <- X.rma.full.invgeo$beta + rep(X_ranef_invgeo$study.ID$
    intrcpt, rep_vec$n) + X_ranef_invgeo$`study.ID/obs`$intrcpt
```

And the corresponding confidence intervals:

```
adjusted_effects_invgeo_se <- X.rma.full.invgeo$se + rep(X_ranef_invgeo$study.ID$se,
     rep_vec$n) + X_ranef_invgeo$`study.ID/obs`$se
```

To then calculate the distance matrix for the adjusted effect sizes:

```
ad_effect_dist_mat_invgeo <- as.matrix(dist(adjusted_effects_invgeo))
```

And finally, the similarity matrix:

```
ad_effect_sim_mat_invgeo <- 1 - (ad_effect_dist_mat_invgeo/max(ad_effect_dist_mat_
    invgeo))
```

Which is then used to get our new, adjusted correlogram:

```
ad_mantel_corlog_geodist <- ncf::mantel.correlog(X_geo_dist_mat_full, ad_effect_sim_
    mat_invgeo, increment = 1, resamp = 0)
```

We can plot this in the same way as the raw correlogram above. The result is, again, shown in Figure 2 (d). This way we can assess whether the removal of correlation between paper proximity and effect size similarity was successful.

D Spurious edges

1560 networks of sizes between 20 and 400 papers, and small (4) and large (12) bias sizes were simulated to assess the influence of different numbers of spurious edges. This was done to assess the potential influence of homonymous author names on the method's performance. Resulting effect size differences were small in all cases an negligible for any realistic number of spurious edges (Fig. 7). For code see supplementary material SC 1 in appendix E.

E Supplementary material

The listed files are available at: https://doi.org/10.5281/zenodo.17712269

Supplementary code

SC 1 Simulation of Networks

SC 2 Reanalysis of Bakdash 2021

SC 3 Reanalysis of Besson 2016

SC 4 Reanalysis of Dinu 2017

SC 5 Reanalysis of Gibson 2011

SC 6 Reanalysis of Moura 2021

SC 7 Reanalysis of Chen 2014

SC 8 Reanalysis of Kredlow 2016

SC 9 Reanalysis of Mathie 2017

SC 10 Reanalysis of Munkholm 2016

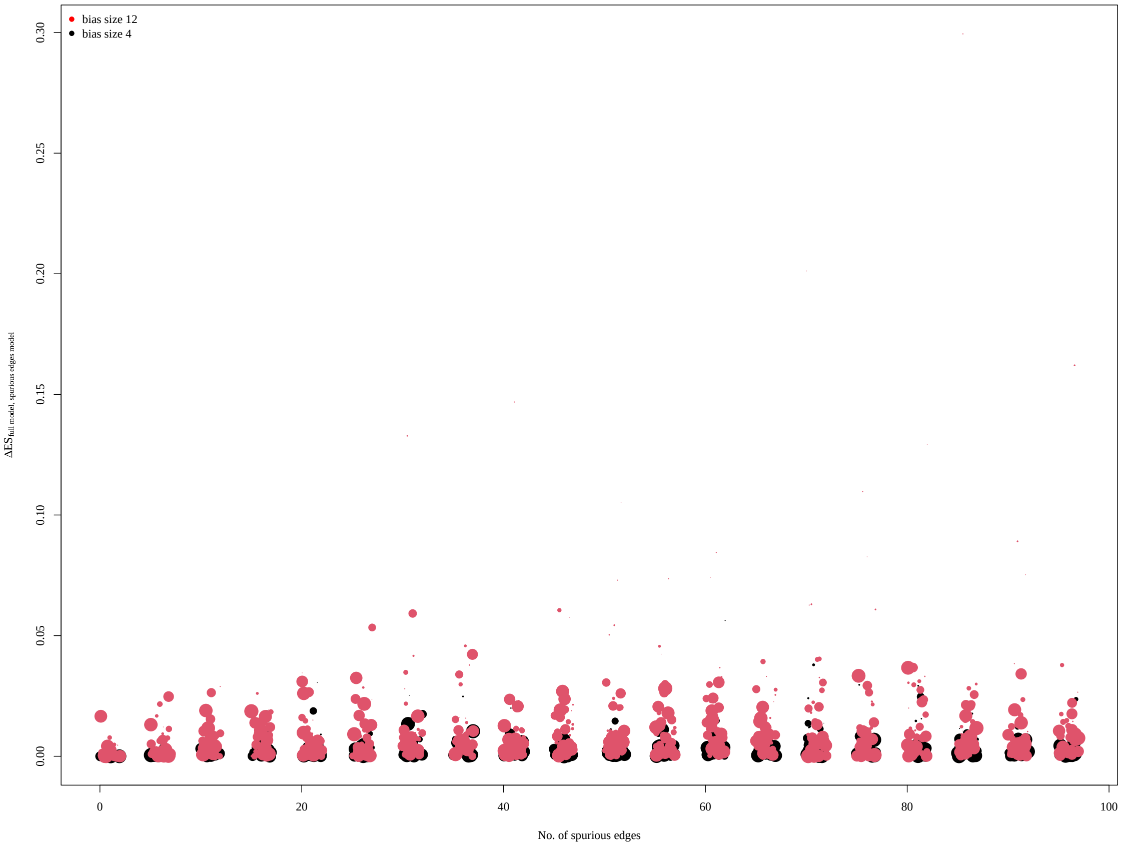

Figure 7: Influence of spurious edges on model performance. y-axis shows the difference in model output between a standard model accounting for authorship bias and a model that was run on additional spurious edges. Red dots show results for large bias, black dots for small bias. Dot size reflects network size. For each level of spurious edges 78 networks were simulated and analysed. Total number of networks n = 1560.

Supplementary data

SD 1.1 Files used for plots in SC 1

SD 1.2 Files used for plots in SC 1

SD 2.1 References of Bakdash 2021

SD 2.2 References of Bakdash 2021 prepared

SD 3.1 References of Besson 2016

SD 4.1 Data analysed in Dinu 2017

SD 4.2 References of Dinu 2017

SD 5.1 References of Gibson 2011

SD 5.2 Data analysed in Gibson 2011

SD 6.1 References of Moura 2021

SD 7.1 Data analysed in Chen 2014

SD 7.2 References of Chen 2014

SD 8.1 Data analysed in Kredlow 2016

SD 8.2 References of Kredlow 2016

SD 9.1 Data (1) analysed in Mathie 2017

SD 9.2 Data (2) analysed in Mathie 2017

SD 9.3 References of Mathie 2017

SD 10.1 Data analysed in Munkholm 2016

SD 10.2 References of Munkholm 2016

References

[1] Bakdash Jonathan Z., Marusich Laura R., Cox Katherine R., Geuss Michael N., Zaroukian Erin G., Morris Katelyn M.. The validity of situation awareness for performance: A meta-analysis *Theoretical Issues in Ergonomics Science.* 2021;23:221–244.

[2] Besson Anne A., Lagisz Malgorzata, Senior Alistair M., Hector Katie L., Nakagawa Shinichi. Effect of maternal diet on offspring coping styles in rodents: A systematic review and meta-analysis *Biological Reviews.* 2015;91:1065–1080.

[3] Dinu Monica, Abbate Rosanna, Gensini Gian Franco, Casini Alessandro, Sofi Francesco. Vegetarian,

vegan diets and multiple health outcomes: A systematic review with meta-analysis of observational studies *Critical Reviews in Food Science and Nutrition.* 2017;57:3640–3649.

[4] Gibson Luke, Lee Tien Ming, Koh Lian Pin, et al. Primary forests are irreplaceable for sustaining tropical biodiversity *Nature.* 2011;478:378–381.

[5] Rios Moura Rafael, Oliveira Gonzaga Marcelo, Silva Pinto Nelson, Vasconcellos-Neto João, Requena Gustavo S.. Assortative mating in space and time: Patterns and biases *Ecology Letters.* 2021;24:1089–1102.

[6] Chen Ying-Ren, Hung Kuo-Wei, Tsai Jui-Chen, et al. Efficacy of Eye-Movement Desensitization and Reprocessing for Patients with Posttraumatic-Stress Disorder: A Meta-Analysis of Randomized Controlled Trials *PLoS ONE.* 2014;9:e103676.

[7] Kredlow M. Alexandra, Unger Leslie D., Otto Michael W.. Harnessing reconsolidation to weaken fear and appetitive memories: A meta-analysis of post-retrieval extinction effects. *Psychological Bulletin.* 2016;142:314–336.

[8] Mathie Robert T., Ramparsad Nitish, Legg Lynn A., et al. Randomised, double-blind, placebo-controlled trials of non-individualised homeopathic treatment: systematic review and meta-analysis *Systematic Reviews.* 2017;6.

[9] Munkholm K, Vinberg M, Kessing L V. Peripheral blood brain-derived neurotrophic factor in bipolar disorder: a comprehensive systematic review and meta-analysis *Molecular Psychiatry.* 2015;21:216–228.

[10] R Core Team . *R: A Language and Environment for Statistical Computing.* R Foundation for Statistical ComputingVienna, Austria 2023.

[11] Oksanen Jari, Simpson Gavin L., Blanchet F. Guillaume, et al. *vegan: Community Ecology Package* 2022. R package version 2.6-4.

[12] Wickham Hadley, François Romain, Henry Lionel, Müller Kirill, Vaughan Davis. *dplyr: A Grammar of Data Manipulation* 2023. R package version 1.1.2.

[13] Wickham Hadley. *stringr: Simple, Consistent Wrappers for Common String Operations* 2022. R package version 1.5.0.

[14] Bjornstad Ottar N.. *ncf: Spatial Covariance Functions* 2022. R package version 1.3-2.

[15] Csárdi Gábor, Nepusz Tamás, Traag Vincent, et al. *igraph: Network Analysis and Visualization in R* 2024. R package version 2.0.1.1.

[16] Carlen Jane. Creating Co-Author Networks in R Retrieved [26.01.2024] from https://datalab.ucdavis.edu/2019/08/27/creating-co-author-networks-in-r/ 2019.
